# Consumptives and Climate

**Published:** 1873-05

**Authors:** 


### CONSUMPTIVES AND CLIMATE.

EVERY once in a while it is stated that some new place is favorable toc onsumptives; generally it is a new country, and the conclusive argument is that few persons are ever known to die of consumption. The two main reasons will be found to be, that there are few people there to die of anything, and that persons in new countries spend most of the time in the open air in farming occupations.

A few years ago Minnesota was represented as the most favorable spot in all America for consumptive persons. Very soon it was ascertained to be the opinion of prominent medical men, who had been in the State many years, that of all who visited Minnesota for lung troubles, about three out of four either died there, or soon after they returned home. A great deal was said about the climate being cool and dry and bracing. The trouble was, that those who went there for their health could not get any of the climate. The weather was so piercingly cold from November to April, that the only available climate was a stove room, and that would kill a consumptive anywhere, and would fatalize almost any serious disease.

Now California, especially the southern portion of it, is said to bear the palm for consumption cure. There is a great talk about roses and posies and peas and pinks in

January, and then there is the green grass and the fruit blossoms. But what of all that when winter comes every day at noon, making winter clothing essentially necessary in the streets of San Francisco in July and August, and all the other parts of the year preceding July and succeeding August, and so of all other parts of California where the sea breeze can make its way, bringing with it the searching chills, damps and fogs of ocean, making it as cold as twenty degrees to zero in New York.

It is true there are no hot nights in California, no night the year round which does not make a blanket to sleep under comfortable. But during the long summer, in which it never rains a single day, the universal blackness and dreary brownness of the earth and clouds of alkaline dust whirled up from the soil, now dried to powder, make an atmosphere which is very far from being either pure or healthy. The dry foot-walks of any large town or city during winter, and the shady side of the streets in summer, afford greater facilities for curing a majority of ordinary ailments than any of the vaunted elysiums, West or South, as affording invaluable opportunities of air and exercise and mental diversion every day.

